# The Golden Anniversary Banquet

**Published:** 1904-04

**Authors:** 


### Editorial.

### THE GOLDEN ANNIVERSARY BANQUET.

This banquet, in honor of the surviving members of the Class of 1854, took place at the Hotel Bellevue, Philadelphia, February 27, 1904, and has now passed into history, as all things earthly must do, but it is thought that those who participated in it will not, as yet, consign it to oblivion, but will continue to remember it as one of the most interesting experiences of their lives. This seems to have been the general consensus of opinion of those present.

It is reasonable to ask why such should have been the feeling over a banquet, good as that proved itself to be. It was not the menu, for the average man may be fond of eating and drinking, but these pall and are not uncommon experiences. It was not the men who represented the early training in dentistry, for these, worthy as they may have been, simply recalled one period in educational development. It was not the men who were present from many parts of the country, brilliant as they made this occasion; but the real sentiment that animated that gathering and gave it its joyous character was deeper and more impressive than any or all of these, for, it seemed to the writer, that each one present felt that, in celebrating the passage of fifty years, there was a feeling that the most trying period in dentistry had been surmounted and that the dental profession had come out of it all triumphant.

Fifty years ago dentistry, in the estimation of the world, was only one remove from charlatanry. It was making brave efforts to hold a proper place in professional circles. It attempted local and national organizations, but with only moderate success. Its educational institutions were few in number and young in experience. The teaching was excellent and laid the foundation for a broader culture. Everything in dentistry fifty years ago was in the formative period, and no man then had prescience sufficient to even anticipate the progress of the future. It was that future realized, more than the men who represented it, that made this occasion something to be remembered and one in which the experiences of the past and the hopes of the future were all concentrated. The men came from various sections, not only to act as hosts to the five honored, but, so to speak, to break the bread that represented the promised land which, if not yet quite reached, was in sight, and the years of trial, anxiety, and continuous effort were all comprehended to their fullest extent. The pioneer days were practically ended, the ground had been cleared and the fields prepared for the harvest of the future; nor was it difficult to read the results to be secured, through this past, in that future. The prophetic thought is based on that fifty years of intelligent practice; fifty years of educational effort; fifty years of concentrated thought. The fruitage in the next fifty years must progressively bear an intimate relation to the soil from which it sprang.

The banquet was pre-eminently a family gathering. Men from New England shook hands with men of the South, men of the East gloried in the growth of the West, and the home group felt they were honored by the presence of men, of many sections, who represented the highest in dentistry.

Thus the present met the past, and the glories of the latter paled in the brilliant successes of the former. Speeches were made, for a banquet can have no prestige without this formality, but this, through the delightful and tactful management of Professor Darby, was carried through most successfully. It was throughout an ovation, not wholly to men, but to the profession.

When we contemplate the progress of the past fifty years, not alone in our special line of work, but in all that has marked an advance in science and art in the world at large, the mind is lost in the confusion of new and startling discoveries. Things to-day important, and yet so common that they are regarded by the present generation as though they had always been, were then not only unknown, but were not even a desideratum in the world's life. Civilization has lived in that half-century as it has lived in no former period, and it is, therefore, not surprising that dentistry received its largest growth through an influence that pervaded the entire mentality of the period.

When the young men have passed through a similar half-century of work and in a similar celebration, the question may appropriately be asked, What progress has been made since 1904? It is to be hoped that the answer will be returned that, in comparison, the dentistry of 1904 bears no relation to that of 1954 in its ability to meet all the requirements of a scientific profession. We are very prone to think much further progress in dentistry is impossible, but so thought we all when the spring of 1854 saw the graduating class of the Philadelphia College of Dental Surgery launched upon a sea of professional troubles.

The dentists of Philadelphia have reason to thank those of the country at large for their interest and co-operation in making this not simply a banquet, but a mark in the passage of time from which to date other steps in the advancing years. Without this aid the banquet would have lost its best and most inspiring feature.

The men especially honored upon this memorable occasion, were the surviving members of the Class of 1854. As far as known, but five remain:

| | |
|---|---|
| Louis Jack, D.D.S. | W. Storer How, D.D.S. |
| C. Newlin Peirce, D.D.S. | Eri W. Haines, D.D.S. |

James Truman, D.D.S., LL.D.

The committee in charge deserve special mention, for through their earnest and energetic efforts the success of the banquet is mainly due.

| | |
|---|---|
| Edwin T. Darby, M.D., D.D.S. | G. L. S. Jameson, D.D.S. |
| Edward C. Kirk, D.D.S., Sc.D. | J. D. Thomas, D.D.S. |
| R. Hamill D. Swing, D.D.S. | Wilbur F. Litch, M.D., D.D.S. |
| Albert N. Gaylord, D.D.S. | H. C. Register, M.D., D.D.S. |
| Earl C. Rice, D.D.S. | William H. Trueman, D.D.S. |
| I. N. Broomell, D.D.S. | Robert Huey, D.D.S. |
| J. T. Lippincott, D.D.S. | William L. J. Griffin, D.D.S. |
| L. Foster Jack, M.D., D.D.S. | J. Clarence Salvas, D.D.S. |

D. N. McQuillen, D.D.S.

The class that graduated in 1854 from the Philadelphia College of Dental Surgery was as follows:

William Calvert, Pennsylvania.
Horton Bailey, Pennsylvania.
Firman Coar, Pennsylvania.
Alexander G. Coffin, Massachusetts.
E. H. Cogburn, Mississippi.
Benjamin Cohen, Germany.
Samuel W. Frazer, Pennsylvania.
William Gorges, Pennsylvania.
Eri W. Haines, Delaware.

W. Storer How, Maine.
Louis Jack, Pennsylvania.
Bernard T. Laughlin, Pennsylvania.
C. Newlin Peirce, Pennsylvania.
Isaiah Price, Pennsylvania.
David Roberts, Pennsylvania.
John M. Rothrock, North Carolina.
John R. Rubencame, Pennsylvania.
Thomas H. Shaw, Alabama.
James Truman, Pennsylvania.

The Toasts of the evening were upon the following:

"Welcome," Dr. Wilbur F. Litch.
"Class of '54," Dr. James Truman.
"Our College Days," Dr. Louis Jack, Dr. C. N. Peirce, Dr. W. Storer How, Dr. Eri W. Haines.
"The Mother of Colleges," Dr. B. Holly Smith.
"The Veterans of New England," Dr. L. D. Shepard.
"The Pioneers of Dentistry in New York," Dr. S. G. Perry.
"The Ohio College of Dental Surgery," Dr. H. A. Smith.

Those present at the banquet were the following:

Robin H. Adair, Ga.

Geo. Emery Adams, N. J.

Fred'k Amend, Jr., Pa.

W. V. B. Ames, Ill.

E. H. Angle, Mo.

Frank L. Bassett, Pa.

E. A. Bogue, N. Y.

Chas. F. Bonsall, Pa.

W. A. Borden, Pa.

C. M. Bordner, Pa.

Albert P. Brubaker, Pa.

S. P. Cameron, Pa.

William Carr, N. Y.

J. N. Crouse, Ill.

M. H. Cryer, Pa.

M. B. Culver, Pa.

E. T. Darby, Pa.

Geo. D. B. Darby, Pa.

L. W. Darlington, Pa.

John F. Dowsley, Mass.

J. E. Dunwoody, Pa.

J. Endelman, F., Pa.

J. N. Farrar, N. Y.

L. Ashley Faught, Pa.

Frank D. Gardiner, Pa.

A. N. Gaylord, Pa.

E. S. Gaylord, Conn.

Cyrus M. Gingrich, Md.

Clarence J. Grieves, Md.

S. H. Guilford, Pa.

Joseph, Head, Pa.

D. M. Hitch, Pa.

R. H. Hofheinz, N. Y.

F. Holland, Ga.

Robert Huey, Pa.

George E. Hunt, Ind.

Charles S. Jack, Pa.

L. Foster Jack, Pa.

V. H. Jackson, N. Y.

G. L. S. Jameson, Pa.

C. R. Jefferis, Del.

G. F. Jernigan, N. Y.

Victor S. Jones, Pa.

E. I. Keffer, Pa.

E. C. Kirk, Pa.

Edward P. Kramer, Pa.

C. V. Kratzer, Pa.

Louis C. LeRoy, N. Y.

J. A. Libbey, Pa.

J. Edw. Line, N. Y.

E. G. Link, N. Y.

J. T. Lippincott, Pa.

Wilbur F. Litch, Pa.

J. Bond Littig, N. Y.

H. B. McFadden, Pa.

Charles McManus, Conn.

James McManus, Conn.

D. N. McQuillen, Pa.

Louis Meisburger, N. Y.

Geo. G. Milliken, Pa.

Walter H. Neall, Pa.

J. J. Nelson, Pa.

Joseph W. Noble, China.

R. H. Nones, Pa.

A. W. Orr, Pa.

Fred. A. Peeso, Pa.

S. G. Perry, N. Y.

Chas. E. Pike, Pa.

Chas. M. Porter, Pa.

William J. Potter, Pa.

H. C. Register, Pa.

Ralph B. Reitz, N. Y.

Hugo Rettich, N. Y.

M. L. Rhein, N. Y.

Howard E. Roberts, Pa.

J. Clarence Salvas, Pa.

M. I. Schamburg, Pa.

J. H. Schlinkmann, Md.

Howard S. Seip, Pa.

L. D. Shepard, Mass.

N. T. Shields, N. Y.

B. Holly Smith, Md.

Eugene H. Smith, Mass.

G. Marshall Smith, Md.

H. A. Smith, Ohio.

Thos. C. Stellwagen, Pa.

C. S. Stockton, N. J.

Clinton W. Strang, Conn.

R. H. D. Swing, Pa.

W. H. Taylor, Pa.

Ambler Tees, Pa.

John D. Thomas, Pa.

Burton Lee Thorpe, Mo.

William H. Trueman, Pa.

Chas. R. Turner, Pa.

F. T. Van Woert, N. Y.

Geo. W. Warren, Pa.

S. C. G. Watkins, N. J.

William C. Wilson, Pa.

James M. Winner, Del.

J. A. Woodward, Pa.

H. Newton Young, Pa.

The Faculty of the Philadelphia College of Dental Surgery was composed of the following:

Elisha Townsend, M.D., D.D.S., Professor of Operative Dentistry and Dean.

J. D. White, M.D., D.D.S., Professor of Anatomy and Physiology.

Ely Parry, M.D., D.D.S., Professor of Chemistry, Materia Medica, and Special Therapeutics.

Robert Arthur, D.D.S., Professor of the Principles of Dental Surgery.

T. L. Buckingham, M.D., Professor of Mechanical Dentistry.

D. B. Whipple, M.D., Demonstrator of Surgical and Mechanical Dentistry.

This College completed only four sessions, during which time there were sixty-three regular graduates. Among these should be named Dr. James E. Garretson and Dr. J. Foster Flagg. The faculty of the College withdrew, owing to a disagreement with the Board of Trustees, the latter conferring honorary degrees in opposition to the wishes of the faculty. The latter, on withdrawing, organized the Pennsylvania College of Dental Surgery, and the Philadelphia College of Dental Surgery ceased to exist.

Thus closes the history of this banquet and, in part, the history of the Philadelphia College of Dental Surgery. We may give utterance to a sigh of mournful regret that our companions of 1854 are not with us to mark the developments of time and experience, but such is ever the struggle,—the great battle of life. Civilization is based on continued change, life to death, death to life. The departure of the old, the incoming of new vitality. Let us be thankful that a few remain to glory with their fellows in the progress of their loved profession.

